# Reducing inference cost of Alzheimer’s disease identification using an uncertainty-aware ensemble of uni-modal and multi-modal learners

**DOI:** 10.1038/s41598-025-86110-y

**Published:** 2025-02-14

**Authors:** Misgina Tsighe Hagos, Kathleen M. Curran, Brian Mac Namee, for the Alzheimer’s Disease Neuroimaging Initiative

**Affiliations:** 1 Science Foundation Ireland Centre for Research Training in Machine Learning, University College Dublin, Dublin, D04 V1W8 Ireland; 2 School of Computer Science, University College Dublin, Dublin, D04 V1W8 Ireland; 3 School of Medicine, University College Dublin, Dublin, D04 V1W8 Ireland

**Keywords:** Alzheimer's disease, Computer science

## Abstract

While multi-modal deep learning approaches trained using magnetic resonance imaging (MRI) and fluorodeoxyglucose positron emission tomography (FDG PET) data have shown promise in the accurate identification of Alzheimer’s disease, their clinical applicability is hindered by the assumption that both modalities are always available during model inference. In practice, clinicians adjust diagnostic tests based on available information and specific clinical contexts. We propose a novel MRI- and FDG PET-based multi-modal deep learning approach that mimics clinical decision-making by incorporating uncertainty estimates of an MRI-based model (generated using Monte Carlo dropout and evidential deep learning) to determine the necessity of an FDG PET scan, and only inputting the FDG PET to a multi-modal model when required. This approach significantly reduces the reliance on FDG PET scans, which are costly and expose patients to radiation. Our approach reduces the need for FDG PET by up to 92% without compromising model performance, thus optimizing resource use and patient safety. Furthermore, using a global model explanation technique, we provide insights into how anatomical changes in brain regions—such as the entorhinal cortex, amygdala, and ventricles—can positively or negatively influence the need for FDG PET scans in alignment with clinical understanding of Alzheimer’s disease.

## Introduction

A Magnetic Resonance Imaging (MRI) brain scan can show atrophies in brain regions such as the hippocampus and entorhinal cortex, whose atrophy is usually associated with Alzheimer’s disease^1–3^. So, it is standard practice to recommend MRI for patients showing cognitive impairment in Alzheimer’s disease diagnosis^4,5^. MRI, however, has low sensitivity in the earlier stages of Alzheimer’s disease^6,7^. The biochemical changes that precede anatomical abnormalities in Alzheimer’s disease patients can potentially be identified earlier using Fluorodeoxyglucose Positron Emission Tomography (FDG PET) imaging^6,8^. FDG PET uses a fluorodeoxyglucose tracer to measure and visualize glucose metabolism through tracer uptake in tissues. This complementary nature of MRI and FDG PET modalities is often exploited in multi-modal deep learning applications for Computer Assisted Diagnosis (CAD) of Alzheimer’s disease^9,10^ in which MRI and FDG PET brain imaging modalities are used to train deep learning models for Alzheimer’s disease identification^9–14^. 3D Stereotactical Surface Projections (SSP) of FDG PET scans have also been used to convert the 3D brain scan into 2D images leading to better performing Alzheimer’s disease detection models^15^. By merging the two modalities together, multi-modal learning from MRI and FDG PET gives deep learning models the ability to learn useful information from an embedding of both modalities. This approach has been used to discriminate between Alzheimer’s disease patients and cognitively normal groups^11,16–20^.

While multi-modal learning often leads to improved classification performance, for end-users, it comes with an additional cost of the extra modality compared to uni-modal models, which only require a single modality. This is because multi-modal deep learning models assume simultaneous availability of all modalities during model inference, an assumption that does not align with real-world clinical practice. When diagnosing Alzheimer’s disease, clinicians typically order additional tests, such as FDG PET scans, only when initial findings (e.g., from MRI) are inconclusive^4,21,22^. Hence, there remains a notable gap in the current landscape of medical image classification research concerning the integration of MRI and FDG PET scans for the multi-modal deep learning-based identification of Alzheimer’s disease. Specifically, there appears to be a lack of a model that aligns closely with clinical best practices in brain imaging-based Alzheimer’s disease diagnosis by making initial brain imaging diagnostic inferences solely from MRI data^4,21^. Such a model should be able to recognize its diagnostic uncertainty when analyzing uni-modal MRI data. Based on its uncertainty, it should then signal the need for an FDG PET scan to enhance diagnostic accuracy and subsequently incorporate the additional FDG PET scan data to refine and improve its diagnostic inferences in a multi-modal fashion. This approach is theoretically appealing because it mimics the adaptive, evidence-based decision-making process used by healthcare professionals, where the choice of diagnostic tests is dynamically adjusted based on the information available, the specific clinical context and in line with guidelines that recommend FDG PET scans for inconclusive MRI data^4,21,22^. Such a model would not only optimize the use of FDG PET scans, which are expensive and expose patients to a higher level of radiation compared to MRI scans, but also potentially improve diagnostic outcomes by leveraging the complementary strengths of both imaging modalities.

Uncertainty quantification is very important to understand the limits and capabilities of deep learning models^23^. This is particularly true in medical applications, where the cost of incorrect predictions is significant. By avoiding uncertain outputs of a model, we can reduce the number of incorrect predictions^24^. Deep learning model output probabilities, however, are usually not well calibrated^25^, and taking them for granted as uncertainty estimates can result in overconfident predictions^26^.

More computationally expensive Bayesian Neural Networks (BNNs) provide uncertainty estimates by replacing deterministic weight parameters of deep learners with probability distributions^27,28^. An interesting approach to overcoming the infeasible computational cost of BNNs^28^ is to approximate the Bayesian inference of a Gaussian probabilistic model using dropout layers. This is referred to as a Monte Carlo (MC) dropout estimate^29^. In MC dropout, a model that contains dropout layers is first trained. Then during inference dropout continues to be used, which is not usually done. Running this for a number of iterations generates different model probability outputs. The standard deviation of these outputs is then taken as an uncertainty estimate of the model’s prediction. The higher this standard deviation is, the larger the uncertainty. Evidential Deep Learning (EDL)^30,31^ is another approach that explicitly integrates uncertainty with deep learning. In EDL, the activation functions of a neural network’s final layer are replaced with a ReLu activation function, which is then taken as an evidence vector to directly model uncertainty. Although in the context of uni-modal electroencephalography inputs rather than medical imagining, BNNs and MC-dropout have been used for Alzheimer’s disease diagnosis before^32^.

We propose a dynamic multi-modal deep learning framework that learns from MRI and FDG PET data and leverages uncertainty estimates to address the problem of expensive model inference by requesting FDG PET inputs only when they are needed. By quantifying uncertainty, our method assesses the confidence of MRI-based predictions and decides whether an FDG PET scan is warranted or not. By integrating this adaptive approach, we aim to reduce the number of FDG PET scans needed, thereby lowering costs and radiation exposure while maintaining diagnostic accuracy. To the best of our knowledge, this is the first research work that mimics clinical practice in combining MRI and FDG PET by developing a model that can initially perform inference using only MRI, recognize its own uncertainty, and then request an FDG PET scan when necessary to enhance diagnostic accuracy, subsequently performing inference using both modalities. We highlight the following primary contributions of our work:

- We introduce an approach that switches between uni-modal and multi-modal models based on the uncertainty of the uni-modal model for models trained for Alzheimer’s disease detection using MRI and FDG PET brain imaging modalities.
- We empirically demonstrate that the amount of FDG PET scans that are required by multi-modal models can be reduced by as much as 92% without sacrificing performance.
- We provide insights into why certain MRI scans might require FDG PET scans for accurate differentiation between cognitively normal and Alzheimer’s disease.
- We assess the reliability of MC dropout and EDL uncertainty estimates by evaluating them on Out-of-Distribution (OOD) data.
- We make our code and trained models publicly available to facilitate reproducibility and support future research.

## Methods

In this section, we describe how EDL is used to estimate uncertainty and how we use EDL and MC dropout to build an uncertainty-based multi-modal model. We then describe how the data used in our experiments is collected and prepared, before discussing the model architecture, training, and evaluation techniques.

### Evidential deep learning

EDL uses a Dirichlet distribution with parameters $\alpha _{k}$ ($k = 1, \ldots , K$ where *K* is the number of classes) to calculate uncertainty mass at the final layer of a neural network^30,31^. The uncertainty mass of arguments or subjective opinions in subjective logic represents “second-order uncertainty”^33^. While for each input sample a neural network assigns class label probabilities that are referred to as “first-order uncertainty”, the Dirichlet distribution is the probability density function over the first-order probabilities and expresses second-order uncertainty^33–35^. Assuming we have *K* class labels, this is done by assigning a belief mass $b_{k}$ for each class (for $k = 1, \ldots , K$) and an overall uncertainty mass of *u*. $b_{k}$ and *u* are non-negative and sum to one, $u + \sum _{k=1}^{K}b_{k} = 1$, and the belief mass $b_{k}$ for each class label is computed using the evidence for the class label. For evidence $e_{k} \ge 0$ for class *k*, the belief and uncertainty are computed as,

1 $$\begin{aligned} b_{k} = \frac{e_{k}}{S}, \hspace{0.7cm} u = \frac{K}{S} \end{aligned}$$

where $S = \sum _{k} (e_{k} + 1)$ and zero total evidence means the belief assigned to each class label is zero and leads to total uncertainty, i.e. $u=1$.

Now, let us go back to the relation between the Dirichlet distribution and belief masses. The EDL model outputs a Dirichlet distribution in $K-1$ dimensions. A Dirichlet distribution $P = (p_{1}, p_{2},\ldots ,p_{k})$ with *K* parameters $(\alpha _{k}, k \in {1,\ldots ,K})$ is given by

2 $$\begin{aligned} D(P|\alpha ) = \frac{1}{B(\alpha )}\Biggl \{ {\prod _{k=1}^{K}P_{k}^{\alpha _{k}-1}} \Biggl \} \end{aligned}$$

where $B(\alpha )$ is a K-dimensional multinomial beta function. The Dirichlet distribution parameters $\alpha _{k}$ are obtained from the evidence $e_{k}$ for each class label from the neural network using $\alpha _{k} = e_{k} + 1$. Then, Equation 1 relates the Dirichlet distribution parameters and the belief masses as follows,

3 $$\begin{aligned} b_{k} = \frac{e_{k}}{S} = \frac{e_{k}}{\sum _{k} (e_{k} + 1)} = \frac{\alpha _{k} - 1}{\sum _{k} \alpha _{k}} \end{aligned}$$

Equation 3 means we can obtain a subjective opinion, belief $b_{k}$ and uncertainty *u*, from the parameters of the corresponding Dirichlet distribution. The likelihood function using the sum-of-squares loss^30^ is used to train a network using EDL:

4 $$\begin{aligned} \mathcal{L}_{i}(\Theta ) = \int ||y_{i}-p_{i}||_{2}^{2}\frac{1}{B(\alpha )}\prod _{j}p_{ij}^{\alpha _{ij}-1}dp_{i} = \sum _{j}\textbf{E}[y_{ij}^{2} - 2y_{ij}p_{ij} + p_{ij}^2] \end{aligned}$$

where $p_{k}$ represents the probabilities for class *k* and $y \in \{0, 1\}$.

### Uncertainty-based ensemble of uni-modal and multi-modal models

**Fig. 1:**
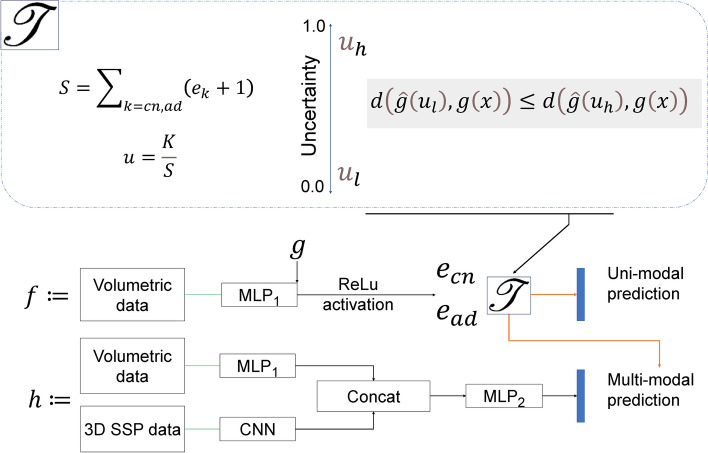
High-level overview of the proposed approach using evidential deep learning (EDL). *cn* and *ad* stand for the categories *cognitively normal* and *Alzheimer’s disease*, respectively.

Figure 1 shows a high-level overview of our proposed uncertainty-based uni-modal and multi-modal ensembling approach using EDL. Here, *f* is a uni-modal MLP model trained to perform both classification of Alzheimer’s disease and identification of uncertain instances to be referred to the multi-modal model, *h*. The multi-modal model, *h*, is composed of both MLPs and a Convolutional Neural Network (CNN). As shown in Fig. 1, identification of uncertain instances is performed with the help of a threshold function, $\mathcal{T}$. $\mathcal{T}$ is implemented in two ways:

1. Simple thresholding that searches for an optimal uncertainty threshold and switches to multi-modality when the uncertainty estimate of the uni-modal model exceeds this;
2. Distance-based thresholding that compares embedding similarities of test instances with a low uncertainty embedding category, $u_{l}$, and a high uncertainty embedding category, $u_{h}$, where the uncertainty cutoff to create both categories is found empirically.

In distance-based thresholding, embeddings of training instances with the lowest and highest uncertainty are collected at the penultimate layer of *f*, which we refer to as *g*, to be used in the threshold function, $\mathcal{T}$. Then, the average of the training instance embeddings belonging to both categories is computed, and we have $\hat{g}(u_{l})$ and $\hat{g}(u_h)$ for the average embeddings of the instances with low and high uncertainties, respectively. When using embedding similarities, at its core, $\mathcal{T}$ does the following comparison:

5 $$\begin{aligned} y = \left\{ \begin{array}{ c l } f(x_{1}) & \quad \text {if } d(\hat{g}(u_{l}), g(x)) \le d(\hat{g}(u_{h}), g(x)) \\ h(x_{1}, x_{2}) & \quad \text {otherwise} \end{array} \right. \end{aligned}$$

where *d* is a cosine similarity, $\hat{g}(u_{h})$ is the average of the embeddings of instances with the highest uncertainty, $\hat{g}(u_{l})$ is the average of the embeddings of instances with the lowest uncertainties, and $x_{1}, x_{2}$ are multi-modal features of the same instance *x*.

$\mathcal{T}$ creates a new uncertainty-based ensemble of uni-modal and multi-modal learners that leverages the strengths of both the uni-modal, *f*, and the multi-modal, *h*, models. We experiment with both EDL and MC dropout estimates to quantify the uncertainty required by $\mathcal{T}$.

### Data preparation

The data used in our experiments was obtained from the Alzheimer’s Disease Neuroimaging Initiative (ADNI) database. ADNI was launched in 2003, led by Principal Investigator Michael W. Weiner, MD (adni.loni.usc.edu). For up-to-date information, see www.adni-info.org.

To create models that are easy to interpret, we use a tabular volumetric data version of $T_{1}$-weighted MRI scans and we use 2D image projections of FDG PET scans. Volumetric MRI data demonstrated strong performance in identifying Alzheimer’s disease^37–39^. We collected the volumetric data of MRI scans from Ledig et al.^36,37^, where the MRI scans were originally collected from ADNI. The MRI scans were segmented using MALPEM with the Neuromorphometrics (http://www.neuromorphometrics.com/) brain atlases, and their structural volumes were extracted^37^. The atlas contains expert delineations of 138 brain regions and so this process results in a tabular data with 138 columns—each column contains the volumetric information of one brain region. The entire list of brain regions can be found as Supplementary Table S1. From the Ledig et al.^36,37^ dataset, we select the volumetric information of 408 participants based on the availability of a corresponding FDG PET scan that was collected within the same year as the MRI scan in the ADNI dataset. Participants’ age is 75.11± 6.89, and the biological sex distribution is 181 females and 227 males.

**Fig. 2:**
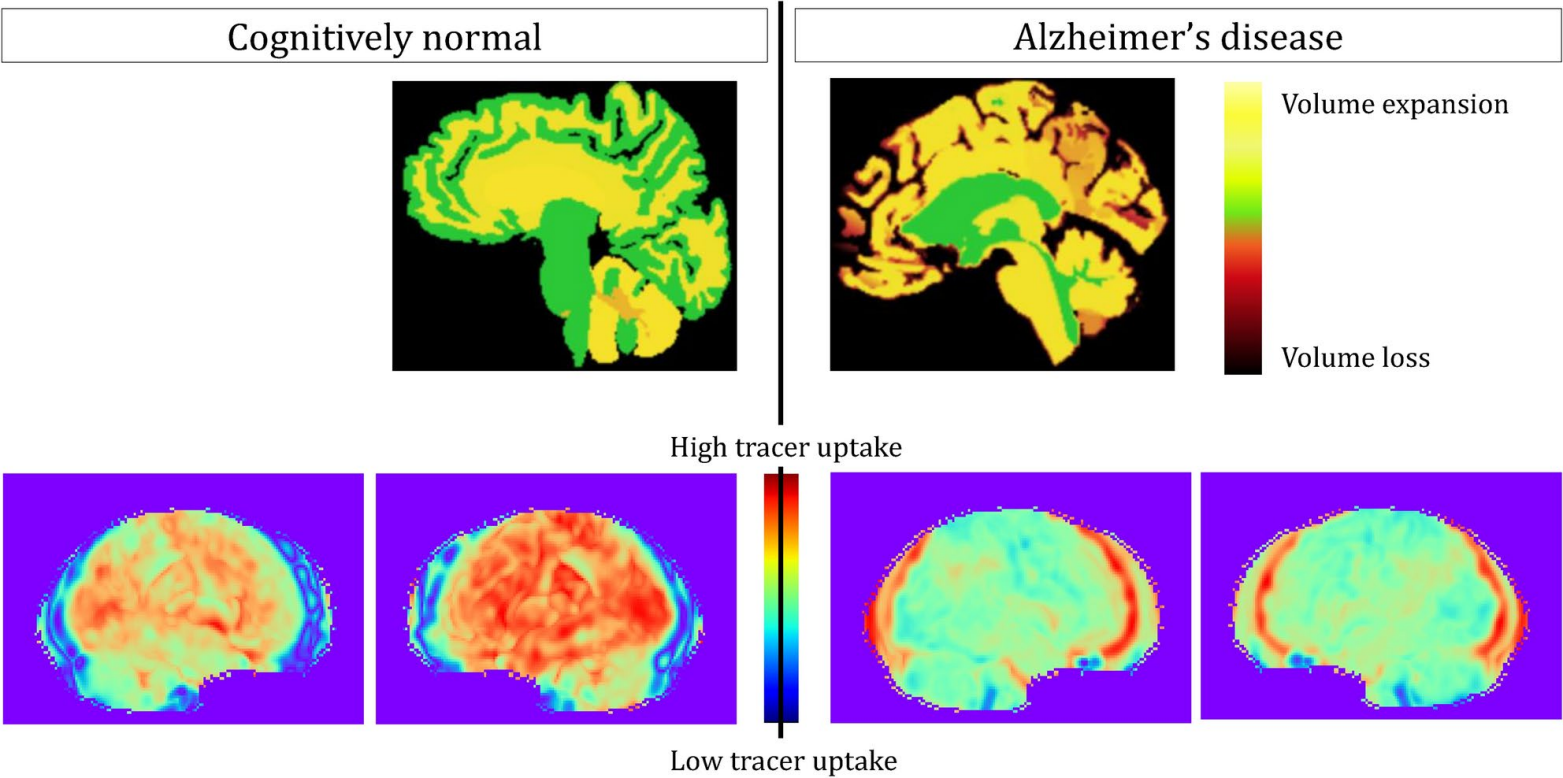
Sample MRI slices (top) and left lateral and right lateral 3D stereotactical surface projections (SSP) of FDG PET (bottom). For presentation purposes, the sample sagittal MRI slices (top) are colour-coded to show volumetric changes compared to 20 cognitively normal control participants. The left lateral and right lateral SSP of FDG PET scans (bottom) are colour-coded to show Fluorodeoxyglucose tracer uptake. The MRI slices are regenerated from^36^ with the authors’ permission.

From the FDG PET scans, we generated 3D Stereotactic Surface Projections (3D-SSP). 3D SSP summarizes 3D brain scans to 2D images by projecting them to predefined surfaces and comparing them against healthy control groups^40,41^. Among the projections, we selected the right lateral and left lateral 3D-SSP of the FDG PET brain scans, as these demonstrated superior performance in previous studies^15^. Sample MRI slices (visualized as volumes change) and samples from 3D SSP of the FDG PET scans from both the cognitively normal and Alzheimer’s disease categories are shown in Fig. 2. The right lateral and left lateral 3D-SSP images in Fig. 2 are colour-coded to show Fluorodeoxyglucose tracer uptake of different brain regions, where red shows a healthy metabolism or high tracer uptake, and green and blue indicate hypometabolism or a lower tracer uptake.

For training and testing models we used the tabular volumetric data, which is of size 408x138, and the right and left lateral SSP projection images, which are each of size 224 × 224 × 3. 180 of the instances were used for model training, and the remaining 228 instances were used for testing.

### Model architecture and training

We implemented the models in all of our experiments using Tensorflow and Keras (https://www.tensorflow.org/api_docs/python/tf/keras). After comparing performances of existing baseline models on the tabular data, we employed KerasTuner (https://keras.io/keras_tuner/) to search for the best-performing architectures and hyper-parameters (the classification performance comparison against baseline models is presented in Supplementary Table S2; in addition to neural networks and support vector machines, we compare performance against variants of ensemble decision trees because these models have been seen to perform well on tabular data^42,43^.) The full architecture of the multi-modal model can be found in Supplementary Figure S1. Based on our architecture and hyperparameter search using KerasTuner, we employ MLPs and CNNs with residual layers. The uni-modal model shares a subset of the multi-modal architecture as shown in Fig. 1 and it contains two dropout layers with a rate of 40%. For the multi-modal models, we used a late-fusion approach where we concatenate embeddings of the separate modalities at the latent space of the models. We used an Adam optimizer with a decaying learning rate of 1e-3 for model training. We used a cross-entropy loss to train the MC dropout based models and 100 forward passes were used to generate MC- based uncertainty estimates. For easier presentation, the MC dropout values are normalized by the highest MC dropout uncertainty estimates in each of the models. When using EDL, the uni-modal and multi-modal model training and uncertainty estimation were performed as described in the “Evidential deep learning” section. We performed all model training on a machine equipped with an Nvidia RTX5000.

### Model evaluation

While we use classification accuracy to assess trained models’ performance, the number of FDG PET scans that are deemed unnecessary when ensembling the uni-modal and multi-modal models is used as a cost reduction metric. The lower the number of required FDG PET scans, the higher the cost reduction. We also evaluate the model uncertainty estimates by assessing their robustness on Out-of-Distribution (OOD) data. The uncertainty estimates on OOD data are expected to be higher than the uncertainty estimates on In-Distribution data (InD) data.

**Table 1:** A summary of accuracy and best cost reduction.

	Model types	Accuracy	Simple thresholding		Distance- based	
			Best cost reduction	Accuracy at best cost	Best cost reduction	Accuracy at best cost
MC dropout	Uni-modal	0.850	**92%**	0.883	54%	0.873
	Multi-modal	0.873				
EDL	Uni-modal	0.851	61%	0.890	42%	**0.903**
	Multi-modal	**0.908**				

The best cost reduction shows the highest percentage reduction of FDG PET scans that were initially required for the multi-modal models.

## Results and discussion

This section presents results and discusses the experiment evaluating the effectiveness of the uncertainty-based merging of unimodal and multimodal models using MC dropout and EDL. Performance and cost gains are discussed. We then present a global model explanation to interpret the reasons for referral for FDG PET. Lastly, the robustness of the uncertainty estimates is assessed on OOD data.

### Impact on accuracy and cost

Table 1 contains a summary of the accuracy and cost gains of our experiments. Without the uncertainty-based thresholding, the results show that with accuracies of 0.873 and 0.908 in the MC-dropout and EDL-based models, respectively, the multi-modal approaches outperform the uni-modal approaches. However, our uncertainty-based merging of the two models surpasses the performance of the uni-modal model and surpasses or achieves comparable results as the multi-modal models while using a much reduced number of FDG PET scans. Among the uncertainty-based merging of uni-modal and multi-modal models, the distance-based thresholding EDL achieves the highest accuracy of 0.903 while reducing the FDG PET use by 42% and the MC dropout simple thresholding achieves the highest FDG PET cost reduction of 92% with an accuracy of 0.883.

**Fig. 3:**
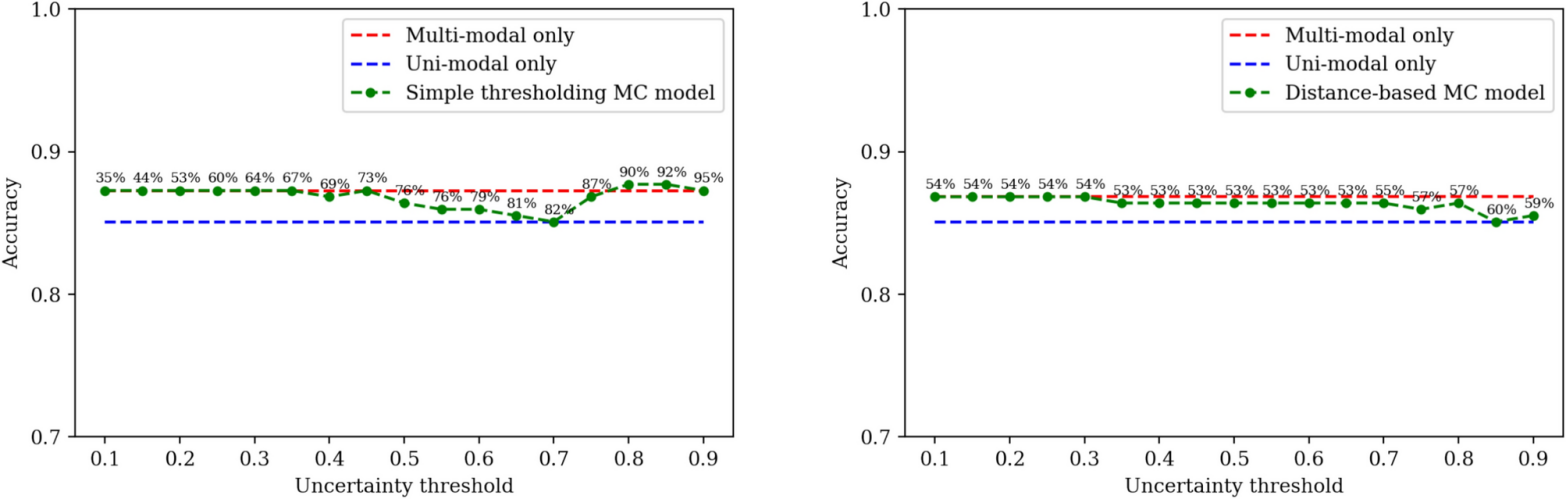
An exploration of model performance for different uncertainty thresholds when Monte Carlo dropout is used to estimate uncertainties. Simple thresholding of uncertainty to choose models using $\mathcal{T}$ (left); Distance-based thresholding of uncertainty to choose models using $\mathcal{T}$ (right). Percentages above the dots represent cost reduction (X% means X% of the FDG PET scans are avoided).

**Fig. 4:**
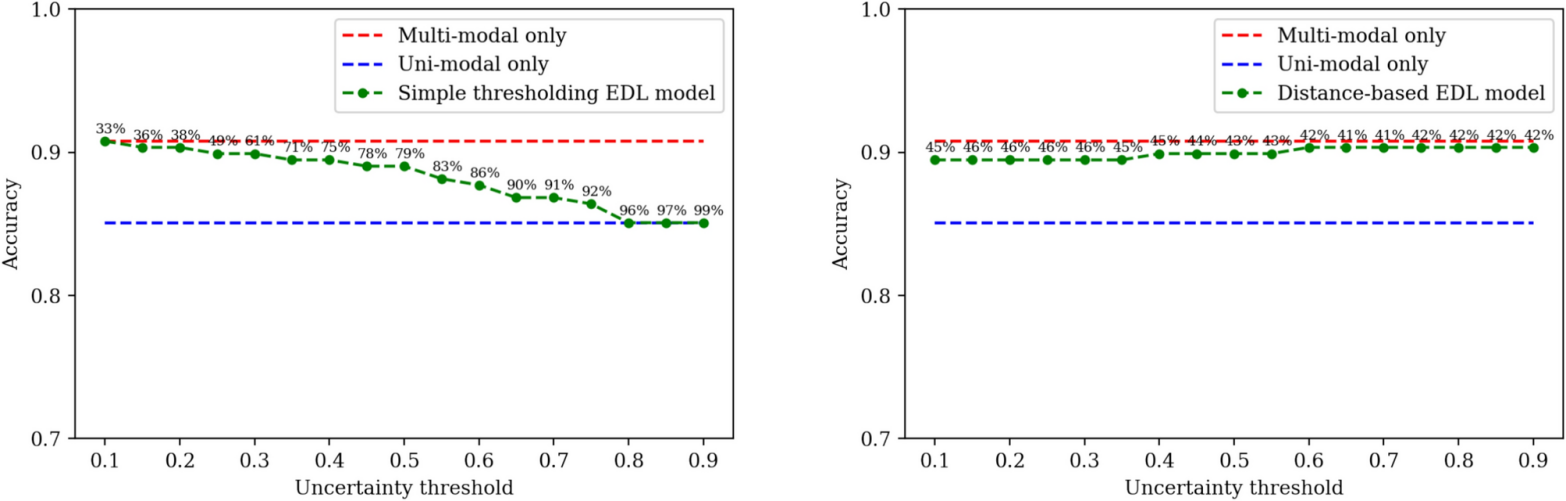
An exploration of model performance for different uncertainty thresholds when Evidential deep learning is used to estimate uncertainties. Simple thresholding of uncertainty to choose models using $\mathcal{T}$ (left); Distance-based thresholding of uncertainty to choose models using $\mathcal{T}$ (right). Percentages above the dots represent cost reduction (X% means X% of the FDG PET scans are avoided).

Figures 3 and 4 contain accuracy and cost comparison along uncertainty thresholds $\in [0.1, 0.9]$. When MC dropout is used to estimate uncertainties, the adaptive approach using simple thresholding outperforms the multi-modal model using just 8% of the FDG PET scans used by the multi-modal model, resulting in 92% cost reduction. In practice, this means the 92% of test participants that would have been ordered for an FDG PET scan did not need it for Alzheimer’s disease diagnosis in the first place. To achieve the same accuracy as the multi-modal model, the simple thresholding of the MC dropout method only needs 27% of the FDG PET scans (73% cost reduction) (see Fig. 3). Similarly, when using the distance-based thresholding with MC dropout uncertainties, FDG PET scan usage is reduced by 54% to reach the same accuracy as the multi-modal model.

The model using simple thresholding of EDL uncertainties reduces FDG PET scan usage by 33% to achieve the same accuracy as the multi-modal model (see Fig. 4). The model using distance-based thresholding of EDL uncertainties reaches a comparable performance with the multi-modal model by reducing PET usage by 42%. This approach also achieved the highest accuracy of 0.903 when compared to the other merging methods.

In summary, our proposed method exploits the best of both the uni-modal and multi-modal models. By avoiding uncertain predictions of the uni-modal model, we get the benefit of picking its best performance. We also have the option to switch to the multi-modal model for the potentially wrong predictions of the uni-modal model.

### Reasons for FDG PET referral

**Fig. 5:**
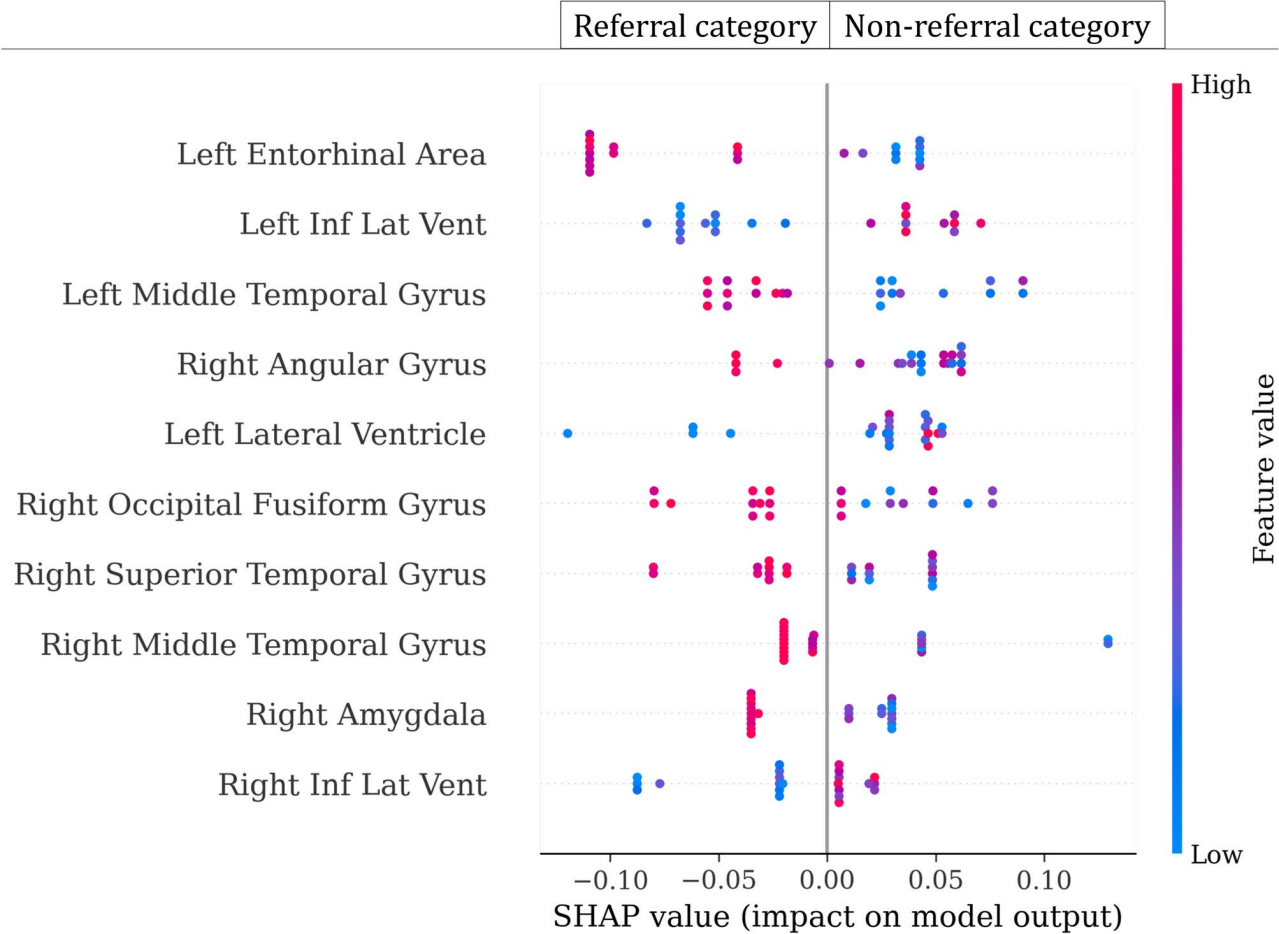
Impact of 10 top-most important brain regions (features) for the non-referral versus referral categories classification. While the MRI scans that were able to be classified with the uni-modal model fall under the non-referral category, the MRI scans that needed FDG PET scans and were thus classified using the multi-modal model fall under the referral category. The positive SHAP value (x-axis) represents the non-referral category, and the negative x-axis represents the referral category.

After identifying the MRI scans that need FDG PET referral from those that do not, the next step is to explain why and see if this reason aligns with our existing knowledge of scenarios where uni-modal MRI is not effective (and where FDG PET is required to make diagnostic decisions). For this section, we use the model with the highest accuracy out of the uncertainty-based models (see the “Results and discussion” section), which is the model using distance-based tresholding of EDL uncertainties. This model reduces the FDG PET referrals that were required for the initial multi-modal model by 42% and achieves a classification accuracy of 0.903. This means that out of the 228 test instances, only 132 would be referred for an FDG PET scan—referral category—and the remaining 96 will only use MRI data—non-referral category. The use of tabular volumetric data puts us in a position where we can train highly performing referral versus non-referral classifier models that can be used to explain why some MRI scans required FDG PET scans and others did not based on volumetric information of brain regions. To do this, we train a Random Forest (RF) model to classify between the referral and non-referral MRI scan categories using a randomly chosen subset of the original test set. We are interested in reducing the size of the RF training dataset because we need to increase the number of instances that will be used to generate explanations. Empirical results showed that 20 instances per category were capable of training the RF model to reach at an acceptable test accuracy of 0.954. The remaining 188 instances were used as a test set for the RF model and to generate model explanations. We then use a global model explanation approach called SHapley Additive exPlanations (SHAP)^44^ to interpret the trained RF model. For brevity, we present the 10 brain regions that were found to be most important by the SHAP explainer in Fig. 5. The explanations shown are based on the volumetric information that exists in the dataset.

Our referral system usually requests FDG PET scans in cases where an MRI is not sufficient. For instance, it has been shown that MRI is critical for identifying ventricular volume expansion^45,46^. For this reason, we would expect ventricular enlargement, shown in the regions right inferior lateral ventricle, left inferior lateral ventricle, and left lateral ventricle (see Fig. 5), to be identifiable with MRI scans and that brain imaging-based Alzheimer’s disease diagnosis of scans with these imaging biomarkers would not be referred for FDG PET scans, but would be made with just an MRI scan. Accordingly, increasing ventricular volume positively affects non-referral. On the other hand, lower ventricular volume positively affects referral because this would be inconclusive to make an Alzheimer’s disease diagnosis.

Another interesting observation is the effect of the left entorhinal area. The entorhinal area is one of the earliest brain regions related to Alzheimer’s disease pathologic changes^1,47^. Therefore, we would expect entorhinal volume loss to be identifiable on an MRI scan. In line with this, smaller entorhinal area volumes are associated with non-referral while larger entorhinal area volumes are associated with referral. This is shown in Fig. 5, where a larger volume of the left entorhinal area (shown in red) positively affects a referral for FDG PET scan as this would appear inconclusive to make a diagnosis decision. The opposite, i.e. lower volume of the brain region, is correlated with the non-referral category.

Reduction of the amygdala volume measured using MRI scan has been proposed as an Alzheimer’s disease diagnostic criterion^48,49^. In agreement with this, as can be seen in Fig. 5, lower volumes of the right amygdala show a correlation with the non-referral category and no need for an FDG PET scan. Overall, we observe the usual patterns of ventricular enlargement or volume loss in the non-fluid regions, such as the entorhinal area and amygdala, leading to non-referral as they are expected to be detected in an MRI scan. The opposite is also true: if an instance contains a small ventricular volume or if the non-fluid regions have large volumes, it is referred for FDG PET scan as these are not common imaging biomarkers of Alzheimer’s disease and can be considered inconclusive. Even though we see similar effects from the temporal gyrus and fusiform gyrus, we did not find conclusive evidence supporting a correlation between their anatomical structure and Alzheimer’s disease in the literature.

### Evaluation of uncertainty estimates

**Fig. 6:**
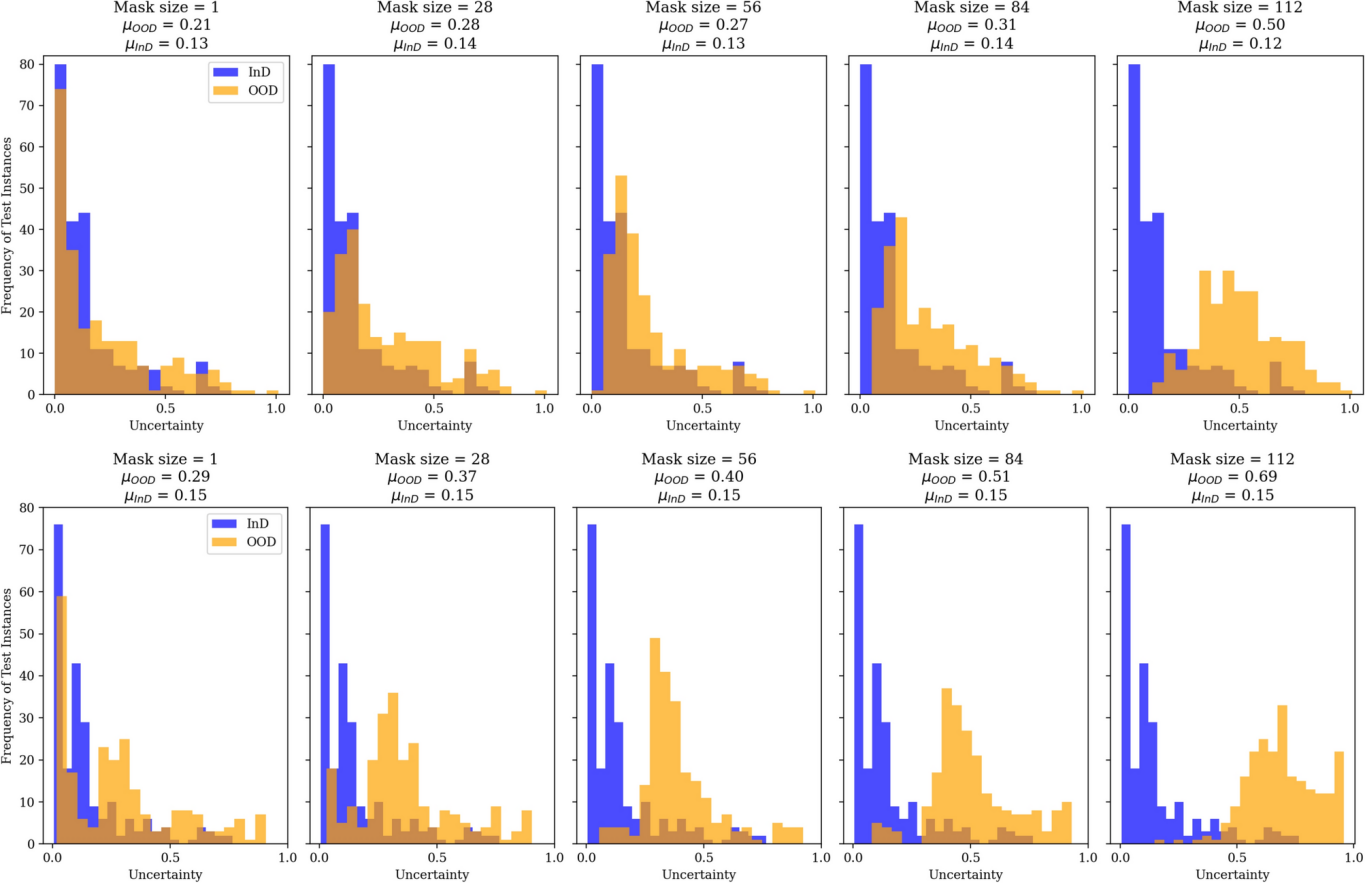
MC dropout (top) and EDL (bottom) uncertainties of in-distribution (InD) () and out-of-distribution (OOD) () uni-modal volumetric MRI data where an overlap is coloured brown. $\mu$ is for the mean of uncertainty estimates. The test dataset of 228 instances is used as InD, and the OOD data are produced from each InD instance by applying the perturbations stated on top of the plots.

**Fig. 7:**
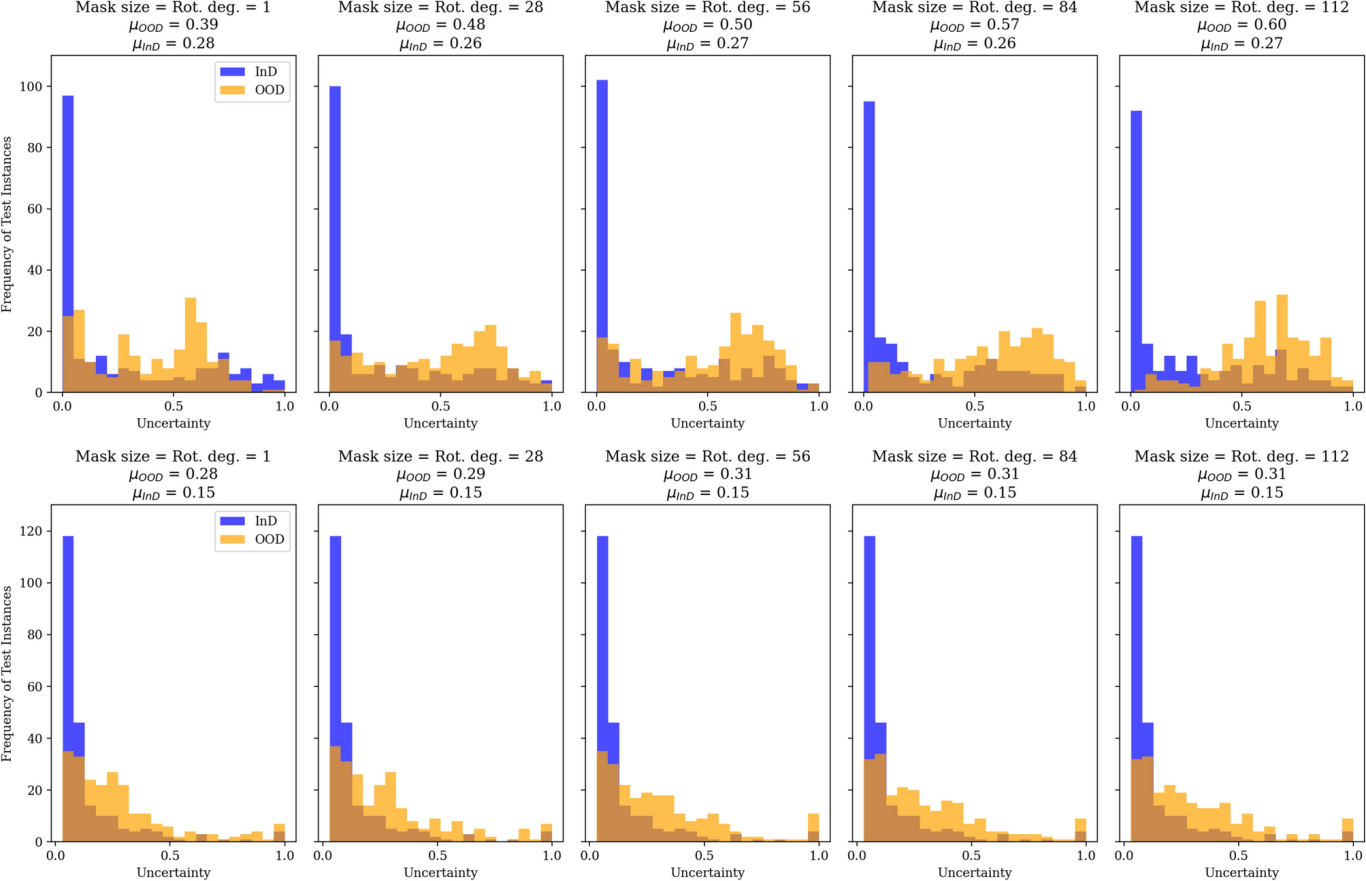
MC dropout (top) and EDL (bottom) uncertainties of in-distribution (InD) () and out-of-distribution (OOD) () multi-modal volumetric MRI and SSP image data where an overlap is coloured brown. $\mu$ is for the mean of uncertainty estimates. Rot. deg. represents the clockwise image rotation degrees. The test dataset of 228 instances is used as InD, and the OOD data are produced from each InD instance by applying the perturbations stated on top of the plots.

Uncertainty estimates are expected to be robust to OOD data, and whenever they are presented with OOD data they should be able to output uncertainty estimates that reflect the level of difference between the input data and their original training data distributions^50^. In other words, they should be able to indicate that “a model knows what it does not know”^51^. To assess the robustness of the uncertainty estimation techniques used in our experiments (MC dropout and EDL), we take inspiration from Sensoy et al.^30^ and Ovadia et al.^51^ to evaluate their performance on OOD data. We generate OOD data by applying masking and image rotation on the InD data, which is the test set of 228 instances. For the volumetric MRI data, we generated OOD data by masking brain regions with zero values. For the multi-modal data, in addition to brain region masking we employed clockwise rotation of the SSP images at different angles. We then compare uncertainties between the InD and corresponding OOD data. We expect increased uncertainty in OOD data when compared to the model uncertainty in InD data. Figures 6 and 7 show uncertainty estimates of MC dropout and EDL on uni-modal and multi-modal InD and OOD data.

As can be seen in Fig. 6, both MC dropout and EDL generate reliable uncertainty estimates when used on the uni-modal OOD data, where we can see a consistent increase in uncertainty as the level of perturbation (masking) on the InD data increases. Coming to the multi-modal OOD data with MC dropout we still see an increase in uncertainty as the perturbation level increases (see Fig. 7). However, it generates exaggerated uncertainty outputs even in reaction to the smallest perturbation; for example, after masking just one brain region and rotating the input SSP images by just one degree, the MC dropout on the multi-modal OOD data outputs a mean uncertainty, $\mu _{OOD} = 0.39$. We continue to see this behaviour as the perturbation level increases. In addition, the uncertainty generated by the EDL for the OOD multi-modal data does not change as much as it did for the uni-modal OOD data (Fig. 7). While our empirical experiments show this behaviour, we leave a theoretical study of why both uncertainties performed better on the uni-modal OOD data for future work. The EDL and MC dropout outputs are not from the same model; thus, we refrain from directly comparing their uncertainty estimates.

## Conclusion

This paper proposes a novel approach to brain imaging-based Alzheimer’s disease detection using a multi-modal model that mimics clinical best practices of FDG PET scan referral of patients. By considering model uncertainty for referral decisions, our approach: (1) reduces the inference cost of multi-modal models by as much as 92%, which eventually reduces patient exposure to higher levels of radiation; and (2) demonstrates how uni-modal models could perform better than, or comparable to, multi-modal models at a much-reduced cost. In addition, since clinicians follow a similar patient referral process when referring for brain scans, we believe it has the potential to ease the adoption of automated systems. The reliability of the uncertainty estimates used is also rigorously evaluated using OOD data.

Due to data availability limitations, we demonstrate the capabilities of our uncertainty-based multi-modality using MRI and FDG PET brain imaging modalities for Alzheimer’s disease identification. However, clinicians might require other imaging and non-imaging modalities and they might be interested in identification of Alzheimer’s disease sub-types. While our evaluation of uncertainty estimates is thorough, we acknowledge the potential for low uncertainty in MRI inputs that may still warrant an FDG PET scan for accurate diagnosis. This limitation underscores the need for a comparative analysis of model uncertainty driven referrals versus expert clinical judgment to better understand and mitigate this gap. Future work should consider these limitations. We have publicly shared our code to support reproducibility and future work.

## Supplementary Information

Supplementary Information.

## Data Availability

The tabular volumetric data of MRI scans were collected from the MALPEM ADNI Data. It can be accessed at the following link: https://gin.g-node.org/ledigchr/MALPEM_ADNI_data. The FDG PET scans were obtained from the Alzheimer’s Disease Neuroimaging Initiative (ADNI) database. For up-to-date information, see www.adni-info.org.

## Code availability

All code and trained models are shared online (link to the repository and trained models: https://github.com/Msgun/uncertainty-based-multimodality).
