# Supplementary material for: Reducing inference cost of Alzheimer’s disease identification using an uncertainty-aware ensemble of uni-modal and multi-modal learners
Source: Sci Rep. 2025 Feb 14;15:5521. doi: 10.1038/s41598-025-86110-y (PMC11828954; doi:10.1038/s41598-025-86110-y)
Supplement: Supplementary file 1 — Supplementary Information. [file 41598_2025_86110_MOESM1_ESM.pdf]

Supplementary Information (Reducing inference cost of Alzheimer's disease identification using an uncertainty-aware ensemble of uni-modal and multi-modal learners)

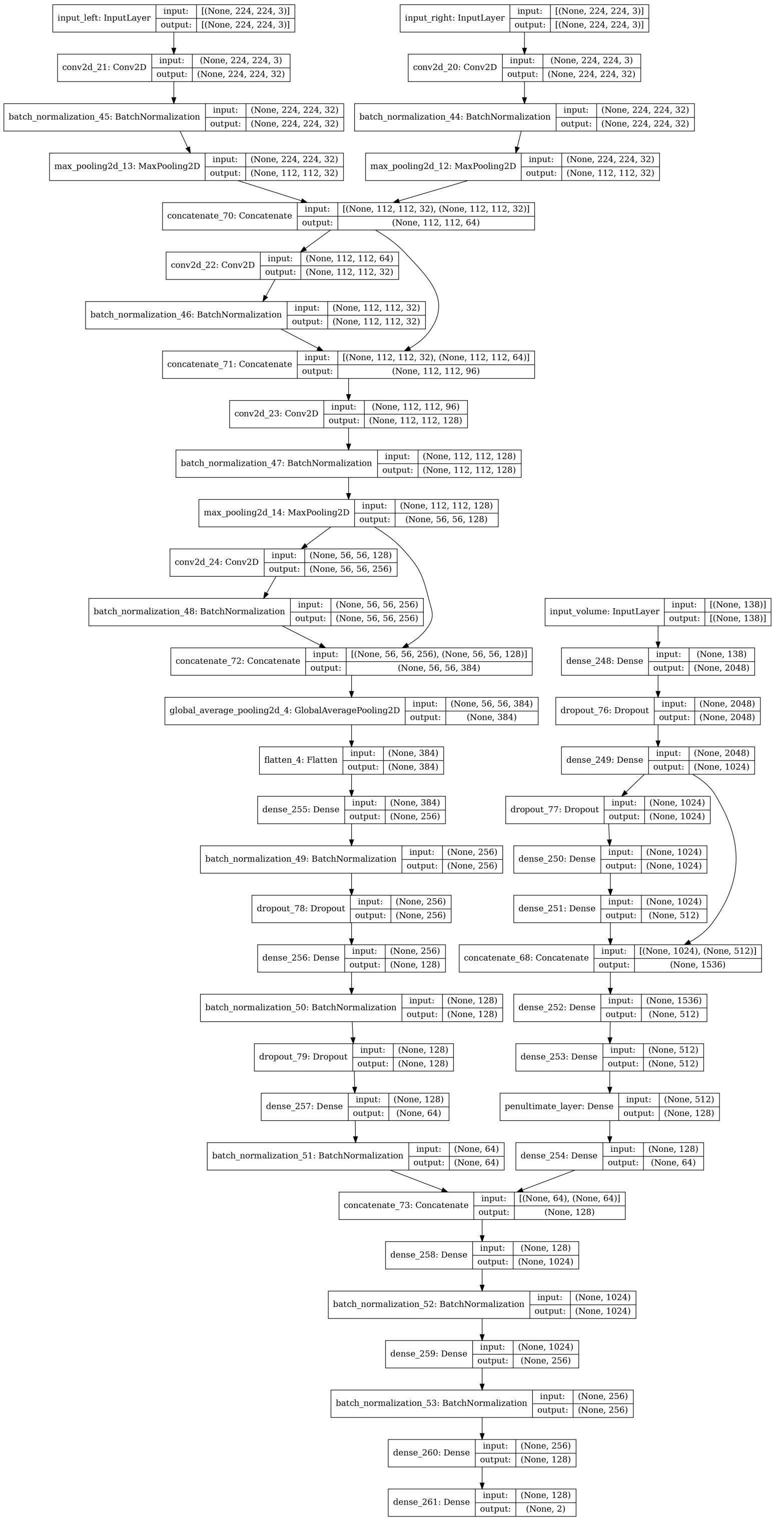

Figure S1. Multi-modal architecture. The uni-modal model uses layers input_volume to dense_254 with its output changed to (None, 2).

Table S1. List of the 138 brain regions whose volumetric information extracted from MRI scans is used to form the tabular dataset. Brain regions were segmented according to the Neuromorphometrics brain atlas (`http://www.neuromorphometrics.com/`).

- Third Ventricle
- Fourth Ventricle
- Right Accumbens Area
- Left Accumbens Area
- Right Amygdala
- Left Amygdala
- Brain Stem
- Right Caudate
- Left Caudate
- Right Cerebellum Exterior
- Left Cerebellum Exterior
- Right Cerebellum White Matter
- Left Cerebellum White Matter
- Right Cerebral Exterior
- Left Cerebral Exterior
- Right Cerebral White Matter
- Left Cerebral White Matter
- Cerebrospinal Fluid
- Right Hippocampus
- Left Hippocampus
- Right Inferior Lateral Ventricle
- Left Inferior Lateral Ventricle
- Right Lateral Ventricle
- Left Lateral Ventricle
- Right Pallidum
- Left Pallidum
- Right Putamen
- Left Putamen
- Right Thalamus Proper
- Left Thalamus Proper
- Right Ventral Diencephalon
- Left Ventral Diencephalon
- Right vessel
- Left vessel
- Optic Chiasm
- Cerebellar Vermal Lobules I-V
- Cerebellar Vermal Lobules VI-VII
- Cerebellar Vermal Lobules VIII-X
- Left Basal Forebrain
- Right Basal Forebrain
- Right anterior cingulate gyrus
- Left anterior cingulate gyrus
- Right anterior insula
- Left anterior insula
- Right anterior orbital gyrus
- Left anterior orbital gyrus
- Right angular gyrus
- Left angular gyrus
- Right calcarine cortex
- Left calcarine cortex
- Right central operculum
- Left central operculum
- Right cuneus
- Left cuneus
- Right entorhinal area
- Left entorhinal area
- Right frontal operculum
- Left frontal operculum
- Right frontal pole
- Left frontal pole
- Right fusiform gyrus
- Left fusiform gyrus
- Right gyrus rectus
- Left gyrus rectus
- Right inferior occipital gyrus
- Left inferior occipital gyrus
- Right inferior temporal gyrus
- Left inferior temporal gyrus
- Right lingual gyrus
- Left lingual gyrus
- Right lateral orbital gyrus
- Left lateral orbital gyrus
- Right middle cingulate gyrus
- Left middle cingulate gyrus
- Right medial frontal cortex
- Left medial frontal cortex
- Right middle frontal gyrus
- Left middle frontal gyrus
- Right middle occipital gyrus
- Left middle occipital gyrus
- Right medial orbital gyrus
- Left medial orbital gyrus
- Right postcentral gyrus medial segment
- Left postcentral gyrus medial segment
- Right precentral gyrus medial segment
- Left precentral gyrus medial segment
- Right superior frontal gyrus medial segment
- Left superior frontal gyrus medial segment
- Right middle temporal gyrus
- Left middle temporal gyrus
- Right occipital pole
- Left occipital pole
- Right occipital fusiform gyrus
- Left occipital fusiform gyrus
- Right opercular part of the inferior frontal gyrus
- Left opercular part of the inferior frontal gyrus
- Right orbital part of the inferior frontal gyrus
- Left orbital part of the inferior frontal gyrus
- Right posterior cingulate gyrus
- Left posterior cingulate gyrus
- Right precuneus
- Left precuneus
- Right parahippocampal gyrus
- Left parahippocampal gyrus
- Right posterior insula
- Left posterior insula
- Right parietal operculum
- Left parietal operculum
- Right postcentral gyrus
- Left postcentral gyrus
- Right posterior orbital gyrus
- Left posterior orbital gyrus
- Right planum polare
- Left planum polare
- Right precentral gyrus
- Left precentral gyrus
- Right planum temporale
- Left planum temporale
- Right subcallosal area
- Left subcallosal area
- Right superior frontal gyrus
- Left superior frontal gyrus
- Right supplementary motor cortex
- Left supplementary motor cortex
- Right supramarginal gyrus
- Left supramarginal gyrus
- Right superior occipital gyrus
- Left superior occipital gyrus
- Right superior parietal lobule
- Left superior parietal lobule
- Right superior temporal gyrus
- Left superior temporal gyrus
- Right temporal pole
- Left temporal pole
- Right triangular part of the inferior frontal gyrus
- Left triangular part of the inferior frontal gyrus
- Right transverse temporal gyrus
- Left transverse temporal gyrus

Table S2. Classification performance of baseline models on the test subset of the tabular volumetric MRI data. Default values are used for the unspecified model parameters.

Model	Parameters	Accuracy
Decision tree	criterion=entropy, min_samples_split=2	0.751
Support Vector Classifier	Regularization parameter (C)=2	0.608
Gradient Boosting Classifier	n_estimators=100, max_depth=10, min_samples_leaf=4	0.808
Random Forest Classifier	n_estimators=100, max_depth=10, min_samples_leaf=4	0.836
TabNet	decision_dim=16, attention_dim=16, n_steps=5, n_shared_glus=2, n_dependent_glus=2, relaxation_factor-1.5, epsilon=1e-15, momentum=0.98, mask_type=softmax, lambda_sparse=1e-4	0.842
XGBoost	booster='gblinear', eta=0.20, subsample=0.6, objective= 'multi:softmax', num_class =2	0.841
